# One-Year Clinical Performance of the Fast-Modelling Bulk Technique and Composite-Up Layering Technique in Class I Cavities

**DOI:** 10.3390/polym13111873

**Published:** 2021-06-04

**Authors:** Louis Hardan, Layla Sidawi, Murad Akhundov, Rim Bourgi, Maroun Ghaleb, Sarah Dabbagh, Krzysztof Sokolowski, Carlos Enrique Cuevas-Suárez, Monika Lukomska-Szymanska

**Affiliations:** 1 Department of Restorative Dentistry, School of Dentistry, Saint-Joseph University, Beirut 1107 2180, Lebanon; louis.hardan@usj.edu.lb (L.H.); rim.bourgi@net.usj.edu.lb (R.B.); maroun.ghaleb@net.usj.edu.lb (M.G.); sarah.dabbagh@net.usj.edu.lb (S.D.); 2 Department of Prosthetic Dentistry, School of Dentistry, Saint-Joseph University, Beirut 1107 2180, Lebanon; layla.sidawi@net.usj.edu.lb; 3 Dental Update Clinic, Mammad Araz Street 102, Baku AZ 1069, Azerbaijan; muradakhundov@mail.ru; 4 Department of Restorative Dentistry, Medical University of Lodz, 251 Pomorska St., 92-213 Lodz, Poland; krzysztof.sokolowski@umed.lodz.pl; 5 Dental Materials Laboratory, Academic Area of Dentistry, Autonomous University of Hidalgo State, Circuito Ex Hacienda La Concepción S/N, San Agustín Tlaxiaca 42160, Hidalgo, Mexico; cecuevas@uaeh.edu.mx; 6 Department of General Dentistry, Medical University of Lodz, 251 Pomorska St., 92-213 Lodz, Poland

**Keywords:** bulk fill, class I, resin composites, nanohybrid composite, polymerization stress

## Abstract

The aim of this study was to assess the one year clinical performance of a new application method, the Fast-Modelling Bulk Technique (FMBT), in comparison to the Composite-Up Layering Technique (CULT) in posterior cavities. Thirty patients with two class I cavities on permanent human molars were enrolled in the present study. A total of sixty class I cavities were prepared and randomly divided according to the restoration technique used: 30 cavities restored by incremental layering technique and modelling of the last layer with Composite-Up Technique (CUT) using the composite Filtek Z250XT (3M ESPE; St. Paul, MN, USA) and the other 30 restored by Bulk Filling technique and modelling of the last layer by Fast-Modelling Technique (FMT) using the composite Filtek Bulk Fill Posterior Restorative (3M ESPE; St. Paul, MN, USA). Restorations were evaluated for up to one year by two observers according to Federation Dentaire Internationale (FDI) criteria, through clinical and radiological exams. Exact Fisher tests were used for statistical analysis. (*p* ≤ 0.05). From a biological perspective, at baseline, teeth restored with both techniques did not reveal any postoperative sensitivity. However, with time, FMBT showed less postoperative sensitivity and therefore more desirable results than CULT with a nonsignificant difference after one year (*p* > 0.05). Concerning secondary caries, fracture of the material, and marginal adaptation, no significant difference was noted between both techniques (*p* > 0.05). Regarding marginal staining, CULT resulted in more staining with a significant difference, as compared to FMBT (*p* < 0.05). Upon radiological examination, FMBT showed a good marginal fit during the first year, whereas CULT showed small empty voids from baseline with a nonsignificant difference (*p* = 1.00). After one year of clinical function, both techniques showed promising results. The present study indicates that the new FMBT could have a positive effect on the marginal staining of resin composite.

## 1. Introduction

Resin composites, classified as synthetic polymers, have dominated dentistry recently. They are composed of dispersed particles of fillers in a resin matrix with catalyst systems and coupling agents. Filler particles mainly consist of quartz, boron silicate, lithium–aluminum silicate glasses, and highly dispersed amorphous silicon dioxide. Special glasses (barium, strontium, and zinc) are added to provide radiodensity. The matrix contains a mixture of various monomers (Bis-GMA, UDMA), comonomers (EGDMA, DEGDMA, TEGDMA), and additives such as photoinitiators (camphorquinone), co-initiators (DMABEE, DEAEMA), inhibitors (BHT), ultraviolet absorbers, photostabilizers, and pigments. The coupling agents (silanes) provide the link between the filler surfaces and (co)monomers. The (radical chain) polymerization of matrix monomers is achieved by opening the double bonds at both methacrylate residues of the monomers resulting in an additive crosslinking [1,2].

Moreover, the rapid progression in resin composite novel technologies through introduction of new products has led to limited clinical data over the years [1]. Meanwhile, the increase in aesthetic demands associated with the development of adhesive techniques make resin composite the best choice for posterior teeth restorations [2]. The optimal performance of resin composite relies on several factors such as: proper polymerization of the resin component, complete isolation, good adhesion, and a good layering technique [3]. Polymerization of the material is always accompanied by a reduction in the volume of the material [4], ranging from less than 1% to up to 6% [5]. Shrinkage can generate stresses that may lead to cuspal deflection, debonding of the restoration, and even enamel microcracks, resulting in subsequent marginal staining, microleakage, postoperative sensitivity, and recurrent caries [6].

Several attempts have been depicted in the literature to solve or control the aforementioned problem [4,7] including, but not limited to: incremental layering technique [2], oblique layering technique, modifying the light activation protocol (soft-start and pulse-delay modes), stress-absorbing intermediate layer (use of thicker nonsolvated adhesive layer, glass ionomer, and flowable composites), modifications in the restorative composite formulation (ring-opening monomers, monomers with higher molecular weight or pre-polymerized additives) [2,4,7,8,9], and recently, the use of bulk-fill composites [10]. Still, there exists some controversy as to which method is considered the optimum in reducing contraction stress [2,3].

Restorative techniques play an important role in stress modification. The Composite-Up Layering Technique (CULT) is the common procedure adopted in clinical practice and was considered a cornerstone in most dental schools worldwide [11]. This procedure optimizes polymerization shrinkage and its internal marginal adaptation using 2 mm thick increments [11,12,13]. Clinically, however, CULT is complex and requires more chair-side time for placing a restoration [14], increasing the risk of presenting voids within increments that can jeopardize the longevity of the restoration [15]. To overcome these problems, recent advances have resulted in replacing the need for incremental layering and focus on the development of composite for bulk placement [16,17], leading to the development of the Fast-Modelling Bulk Technique (FMBT). This technique consists in the application of a bulk-fill resin composite, intended to lower volumetric shrinkage, lower stress of polymerization, increase depth of cure with greater light transmission, and prevent void incorporation and contamination between composite layers [16,18,19,20].

Accordingly, this study aims to compare the clinical performance of the two restorative techniques: the CULT using conventional composite with incremental layering technique, and the FMBT using bulk-fill composite with a single layer. The tested null hypothesis was that the new application method FMBT does not affect the clinical performance over a period of up to 1 year when compared with CULT.

## 2. Materials and Methods

### 2.1. Study Design and Participants

The study was approved by the ethics committee of the University of Saint-Joseph Beirut (FMD-154 #ref: USJ-2017-128). Informed consent was obtained from all individual participants of the study. From February 2019 to May 2020, all adult patients attending the Faculty of Dental Medicine at Saint Joseph University of Beirut who required two class I restorations were asked to participate in the study. A total of 30 patients who met the criteria participated in this study. The mean age of the patients was 25.8 ± 7.49 years with 12 men and 18 women. The inclusion criteria were adult patients with an age range between 18 and 45 years old, requiring two class I restorations in permanent premolars and molars and who could attend the recalls (1 week, 3 months, 6 months, and 1 year). The patients should have good oral hygiene, permanent dentition, class I occlusion, free of any occlusal interferences, the gingival tissue around the tooth to be restored is required to be sound and healthy, good mental condition to fill the informed consent, and to participate in a clinical study. The exclusion criteria include: sensitive teeth, endodontically treated teeth, pregnant women, patients with orthodontic treatment, and bruxism. All patients were informed about the background of the study and the follow-up evaluations. Reasons for placement of the composite restorations were carious lesions, fracture of old amalgam or composite fillings, or replacement for aesthetic reasons.

All patients were treated by one operator. After administering local anesthesia, teeth were cleaned with a rubber cup and fluoride-free prophylaxis paste. Preparation of the cavities was limited to the removal of caries or old insufficient restorations with maximum preservation of sound tooth structure. The internal line was rounded, and the enamel margins were then prepared with butt joint margins. Afterward, cavity preparation was carried out using 80 μm grit cylindrical diamond burs and finished with 25 μm grit cylindrical diamond burs (ISO 012 Intensiv, Viganello-Lugano, Switzerland) under copious water cooling. Next, the operative field was carefully isolated under rubber dam (The Hygenic Corp., Akron, OH, USA). Thereafter, 32% phosphoric acid gel (Scotchbond™ Universal Etchant, 3M ESPE; St. Paul, MN, USA) was applied on enamel that was etched for 30 s, then on dentine for 15 s. Following etching, the cavity was rinsed with water from an air–water syringe for 30 s, until visible moisture or glistening of dentine was observed.

In all cavities, a two-step etch-and-rinse adhesive Adper™ Single Bond (3M ESPE; St. Paul, MN, USA) was applied according to manufacturer’s instructions, and then light-cured with a LED light for 20 s at 1100 mw/cm^2^ (Elipar S10; 3M ESPE; St. Paul, MN, USA). After application of the adhesive, the cavities in each patient were randomly distributed according to the used system: FMBT or CULT.

Manufacturer and composition of the materials used for performing the restorations are listed in Table 1.

The cavities allocated into the CULT group were restored with an incremental layering technique using a conventional composite as follows: the cavity was restored by multiple 2 mm layers of composite Filtek Z250 XT (3M ESPE; St. Paul, MN, USA), leaving 2 mm at the occlusal level to restore the dental anatomy. Each layer was cured for 20 s using a light-curing lamp: Elipar S10 (3M ESPE; St. Paul, MN, USA) at 1100 mw/cm^2^. Restoration and modelling of the last layer by the CUT, following tooth anatomy: each cusp was restored individually and light-cured for 2 s, and lastly, a full photo-polymerization of 20 s was applied for the entire occlusal.

The preparations allocated into the FMBT group were restored by a single layer of a bulk-fill composite as follows: the cavity was restored by a single layer of bulk-fill posterior restorative composite (3M ESPE; St. Paul, MN, USA) with a regular consistency of 4 mm thickness and then light-cured for 10 s per surface using a light-curing lamp Elipar S10.

**Figure 1:**
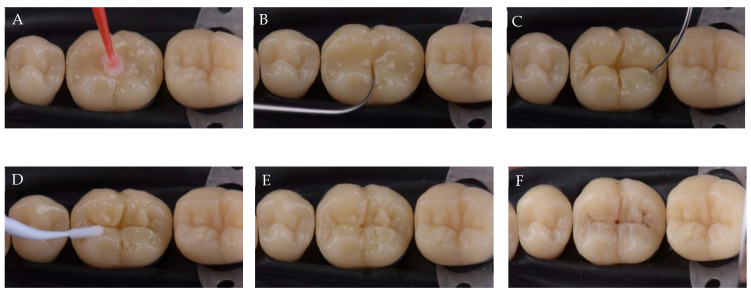
FMBT on class I molar cavity. (**A**) Bulk-fill adaptation using micro-brush; (**B**) first cut of the composite following the remaining fissure with the help of LM Arte fissura; (**C**) drawing the fissures by cutting the composite following the remaining fissures and the anatomy of the teeth; (**D**) cusps were brought together in harmony using a microbrush by reducing the volume of the gap created by cutting the composite; (**E**) stains were applied to give the restoration some aesthetic and 3D appearance; and (**F**) final restoration.

One should keep in mind that if the cavity was deeper than 4 mm, a 2 mm depth should be kept unfilled in order to design the last layer by FMT. This layer was modelled using the same composite. First, with the help of LM Arte fissura (LM-Dental, Parainen, Finland), each cusp is prepared respecting the tooth anatomy. Following the remaining fissures, cusp tilt, marginal ridges, and the remaining marginal fossae, the main grooves were drawn by cutting the composite in order to reduce the C-factor. Next, the excess was removed with the same probe, and the cusps were brought together in harmony using a microbrush to reduce the volume of the gap created while cutting the composite. Finally, the whole restoration was light-cured for 40 s with the same LED lamp (Figure 1). The staining of the final restoration was performed using the LM Arte Fissura (LM-Dental, Parainen, Finland) instrument depending on the patient dentition.

Finishing and polishing of all restorations were performed with cooling underwater using fine-grit diamond burs, polishing discs (Sof-Lex, 3M, St. Paul, MN, USA) and a composite polishing kit (Enhance, Dentsply, Milford, DE, USA). High-gloss polishing was achieved with Prisma^®^ GlossTM polishing paste (Dentsply Caulk, Milford, DE, USA) applied with a foam cup.

**Figure 2:**
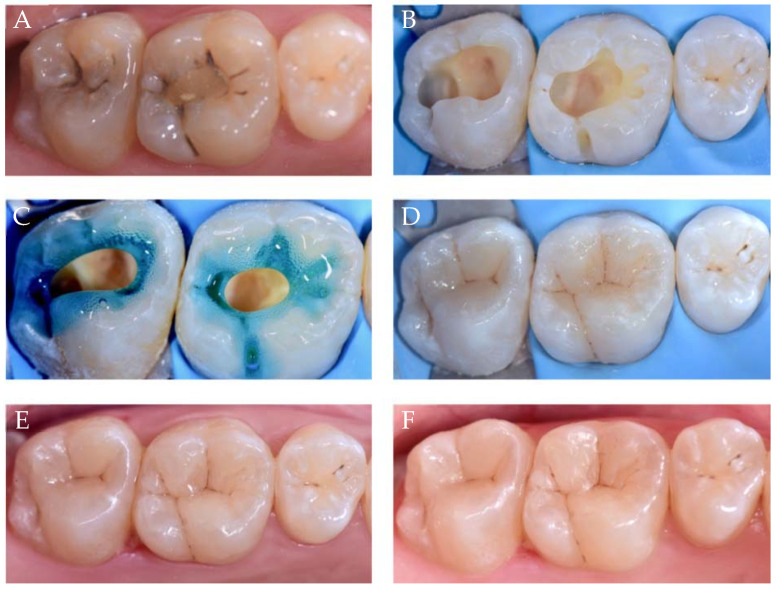
Description of FMBT and CULT on two class I cavities, where 16 shows the FMBT and 17 shows the CULT. (**A**) Preoperative situation; (**B**) cavity preparation and rubber dam placement; (**C**) phosphoric acid applied first on enamel before being applied on dentin; (**D**) FMBT on 16 and CULT on 17, and some stains were applied to give the restoration a 3D effect; (**E**) immediately after finishing and polishing; and (**F**) 1 year follow-up.

Figure 2 shows two decayed teeth (class I cavities in 16 and 17) and the step-by-step clinical preparation and restoration.

### 2.2. Clinical Evaluation

Before the clinical evaluation, patients received a professional teeth cleaning. Restorations were assessed by two evaluators at baseline, 7 days, 3 months, 6 months, and 1 year later according to FDI criteria (Table 2). The evaluation of marginal adaptation, secondary caries, and the composite fracture was assessed by visual inspection using a probe and a mirror. Marginal staining was assessed by visual inspection using a mirror. Moreover, patients were asked to rate the postoperative sensitivity by placing a mark on a visual analog scale (VAS) with units from 0 to 10 (where 0 = no pain and 10 = excruciating pain).

Next, clinically radiographs assessments were taken preoperatively for each tooth to be restored and at each follow-up session. Bite-wing radiographs and clinical photos were taken at the beginning and the end of the restoration, after 1 week, 3 months, 6 months, and 1 year to assess the marginal adaptation of the restorations.

Any discrepancy or variation regarding the part of the scores, and restorations were resolved through discussion and consensus by a third evaluator. The obtained data were recorded using a standardized paper case report form.

### 2.3. Statistical Analysis

Statistical analysis of the data was performed using the Statistical Software package for Social Science (SPSS for Windows, Version 25.0, Chicago, IL, USA). The level of significance was set at a *p* ≤ 0.05. The judgment criteria of the study were biological (sensitivity and secondary caries), functional (fracture, marginal integrity, and radiological adaptation), and aesthetic (marginal staining). These criteria were studied according to time (baseline, 3 months, 6 months, and 12 months) and the nature of the restoration: FMBT and CULT. These are the ordinal variables coded: clinically excellent, good, sufficient, unsatisfactory, and poor. Exact Fisher tests were conducted to compare each of the criteria between the two restorations over time.

## 3. Results

### 3.1. Comparison of Biological Criteria

#### 3.1.1. Sensitivity

**Figure 3:**
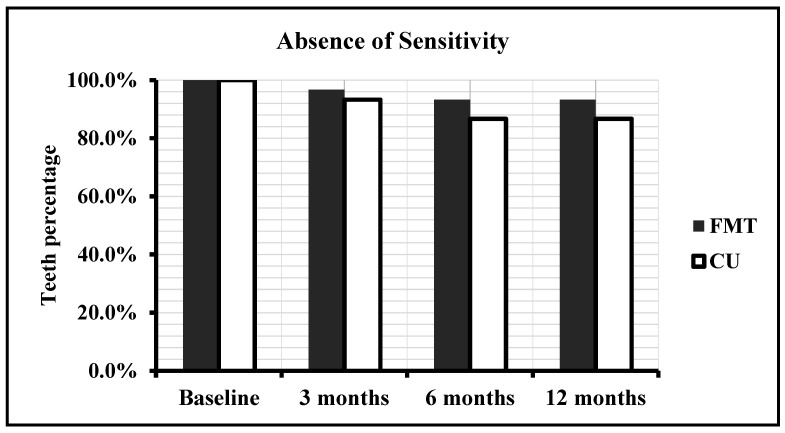
Absence of sensitivity in restored teeth with FMBT and CULT over time.

The number and percentage of teeth restored with CULT and FMBT with regard to postoperative sensitivity are shown in Table 3 and Figure 3. For teeth restored with FMBT, it ranged from 0% up to 6.7%; however, the difference was not significant (*p* = 0.224). For teeth restored with CULT, it ranged from 0% up to 6.7%; however, the difference was not significant (*p* = 0.079). In addition, a statistically significant difference between CULT and FMBT (*p* > 0.05) was not found.

#### 3.1.2. Secondary Caries

**Figure 4:**
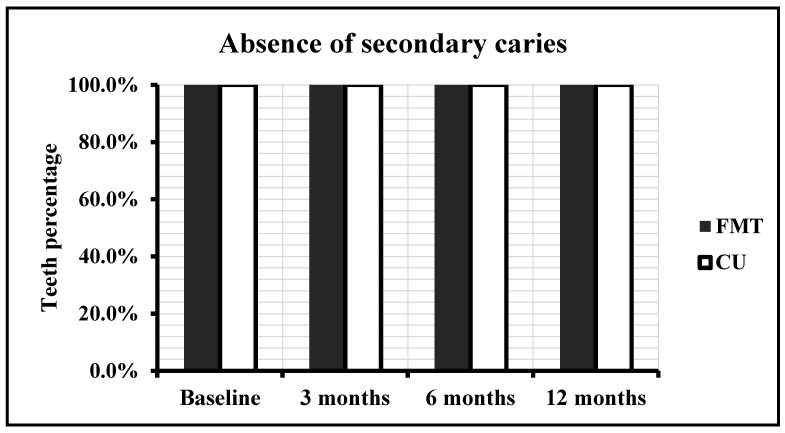
Absence of secondary caries in teeth restored with FMBT and CULT over time.

The number and percentage of teeth restored with CULT and FMBT with regard to secondary caries is shown in Table 4 and Figure 4. This study showed that 100% of FMBT restorations and 100% of CULT restorations showed no signs of caries or signs of demineralization during the 12 months of follow-up.

### 3.2. Comparison of Functional Criteria

#### 3.2.1. Fracture

**Figure 5:**
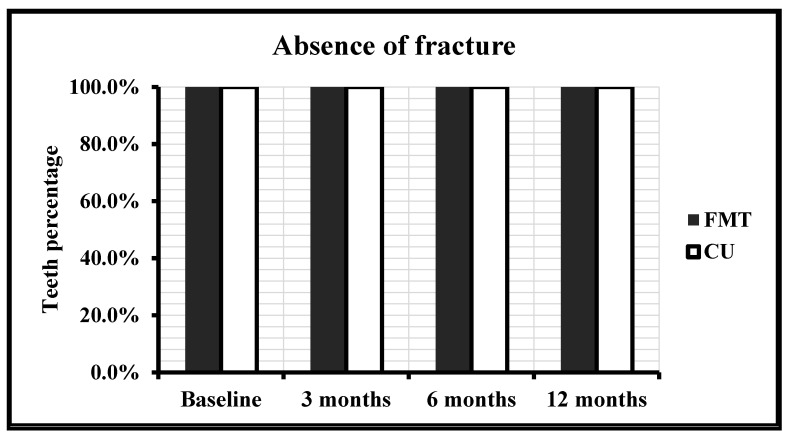
Absence of fracture for teeth restored with FMBT and CULT over time.

Fracture assessment of FMBT and CULT is described in Table 5 and Figure 5. This study showed that 100% of FMBT restorations and 100% of CULT restorations are in place and no fractures occurred within the 12 months post-treatment.

#### 3.2.2. Marginal Integrity

**Figure 6:**
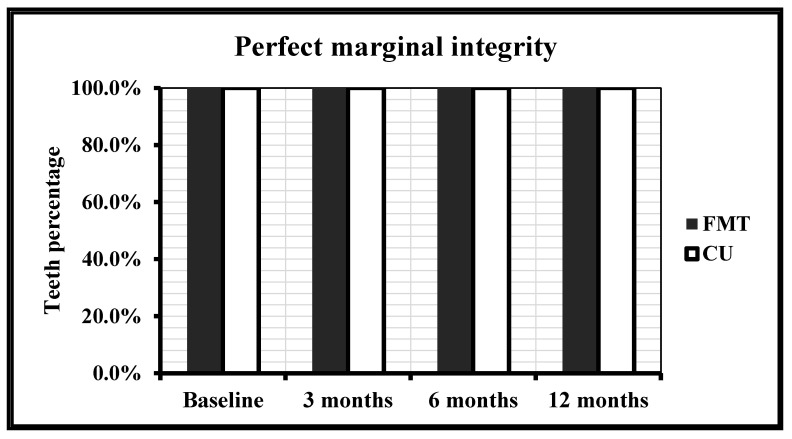
Absence of secondary caries in teeth restored with FMBT and CULT over time.

The evaluation of the number and percentage of teeth restored with FMBT and CULT is presented in Table 6 and Figure 6. This study revealed that 100% of FMBT restorations and 100% of CULT restorations presented perfect marginal integrity during the 12 months post-treatment: a continuity is shown between the restoration and the tooth.

#### 3.2.3. Comparison of Radiological Criteria

##### Marginal Adaptation

**Figure 7:**
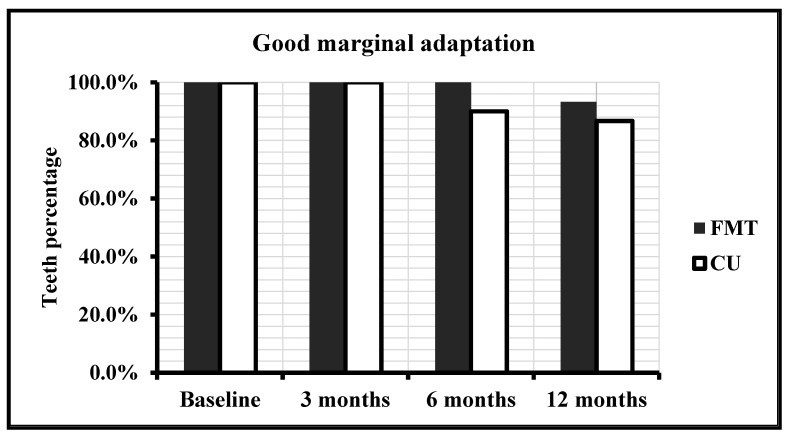
Marginal adaptation observed radiologically for FMBT and CULT over time.

The evaluation of the number and percentage of teeth restored with FMBT and CULT with regard to X-ray marginal evaluation is described in Table 7 and Figure 7. For the teeth restored with FMBT, 100% of the restorations have a marginal adaptation at all follow-up visits (*p* = 1.000). For the teeth restored with CULT, 3.3% presented small gaps between the restoration and the dental walls at baseline and 3 and 6 month follow-up visits; however, the difference was not significant (*p* = 1.000). In addition, a statistically significant difference between FMBT and CULT (*p* > 0.05) was not found.

### 3.3. Comparison of Aesthetic Criteria

#### Marginal Discoloration

**Figure 8:**
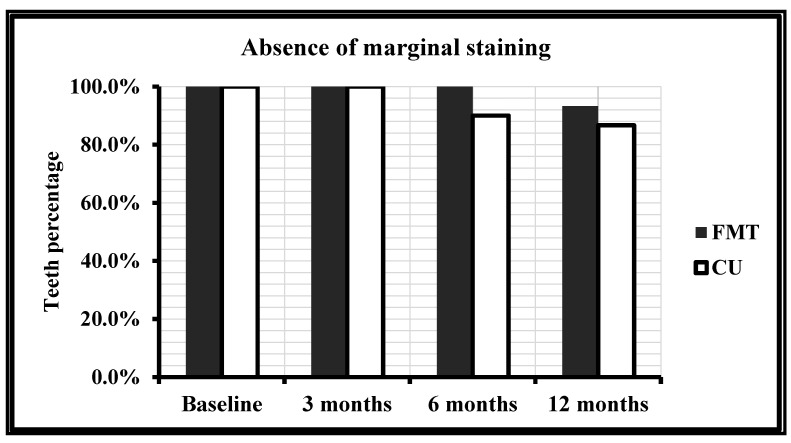
Absence of marginal staining with FMBT and CULT over time.

The evaluation of the number and percentage of teeth restored with FMBT and CULT with regard to marginal staining is presented in Table 8 and Figure 8. Neither of the teeth, which were restored with FMBT, showed any marginal staining overtime at baseline, 3 or 6 months; although 6.7% showed staining at 12 months; however, the difference was not significant between the percentages (*p* = 0.244). Moreover, with CULT, no teeth showed staining at baseline and 3 months, 10.0% showed staining at 6 months, and 13.3% at 12 months; however, the difference was statistically significant (*p* = 0.039). Thus, the occurrence of discoloration was higher with CULT compared to FMBT (*p* < 0.05).

## 4. Discussion

Nowadays, the application of resin composites in posterior cavities is associated with clinical challenges [21]. For a long time, it was considered that incrementally restoring cavity and modelling of the last layer with CUT noticeably reduces shrinkage stress [22]. This procedure guarantees a restoration with good physical properties, good marginal adaptation, and a reduction in cytotoxicity [23].

However, according to Veloso et al. an air entrapment and voids incorporation between different layers of composite can occur [24] resulting in tooth sensitivity and accelerated degradation of the restorative material [25].

Accordingly, several researchers recommended the restoration of the entire cavity with a single layer [10,11]. This can be done with the bulk-fill type of composite material. One of the main problems with this technique is the reduction of polymerization at the deepest part of the material due to the attenuation of light [26]. On the other hand, bulk-fill composite has been the focus of many studies [5,13,16], and recently, the composition of these materials has been altered in various ways by manufacturers [2,5]. They introduced new generations of bulk-fill composites with more powerful photo-initiator systems, allowing increased depth of cure [22] while having decreased volumetric shrinkage and shrinkage stress than previous generations [27].

Meanwhile, the dentist can use these materials in a thick layer of 4–5 mm, simplicity and quick modelling were achieved with better results [16,24]. These resins were then complemented with a final layer of conventional resin composite [28]. The bulk placement with modelling of the last occlusal layer with low-stress composite in a single time according to the tooth anatomy was achieved, by a new technique called FMT. In this sense, this study focused on comparing two systems: the nanohybrid composite with the CUT (old school) and the bulk-fill composite with the FMT (new technique).

The results of the present study demonstrate that regardless of the filling technique CULT or FMBT, all the evaluated clinical parameters: biological (sensitivity and secondary caries) and functional (fracture, marginal adaptation, and radiological) criteria were similar. An exception was for marginal staining where FMBT presents a statistically significant lower discoloration in comparison to CULT. Consequently, the null hypothesis tested was partially accepted.

Despite recent scientific advances in restorative dentistry, postoperative sensitivity remains the common problem when restoring classes I and II cavities [29]. Excessive stress during polymerization is considered the major factor that may lead to sensitivity [4]. In general, sensitivity disappears in the first week after restoring the cavity and tends to decrease over time [30]. However, in some cases, it can persist longer and lead to failure of the restoration [31]. Some studies suggested that postoperative sensitivity after 90 days following class I restoration placement can be related to the stresses generated by polymerization of the resin material at the bonded interfaces and/or by possible accelerated degradation of the adhesive system [32,33]. According to Veloso et al. [24], no association was found between the composite layering technique and the cavity depth on postoperative sensitivity. This finding is in agreement with this study regardless of the technique used, CULT or FMBT; the occurrence of postoperative sensitivity was not observed at baseline. Furthermore, at 3 months, 6 months, and 1 year, the percentage of patients complaining of pain was higher in CULT, and this might be related to the cusp deflection, or adhesive failure [32]. However, the difference between both systems was not significant (*p* > 0.05).

Moreover, the occurrence of postoperative sensitivity is correlated to the complex design of the cavity [33]. In this study, the included class I restorations have the highest configuration factor among all preparations (C-factor = 5), since they have six surfaces, among which the only one is unbonded. It can result in significant shrinkage stress and gap formation [34].

In the randomized clinical trial by Costa et al., postoperative sensitivity compared between both bulk-fill and incremental technique gave almost the same risk and intensity of postoperative sensitivity [35]. This outcome might match the results of this study as the prevalence of tooth sensitivity over time was similar between both techniques. Despite this, many authors suggested that the increase in the amount of resin composite placed in posterior teeth resulted in increased postoperative sensitivity of direct restorations [33,36]. However, unfortunately, a similarity in postoperative sensitivity was witnessed in both techniques FMBT and CULT. One should bear in mind that the lack of postoperative sensitivity was considered a crucial factor in the clinical success of a resin composite restoration. Moreover, it was found that the restoration complexity and the restorative procedure correlate, however insignificantly, with the occurrence of postoperative sensitivity [33]. Although other factors containing patient-related variables, the adhesive system deployed, and the operator were proposed as contributing factors for postoperative sensitivity [34,37,38]. However, the age of the patients participating in this study was between 18 and 45 years old. This narrow range is considered for standardization. In addition, the same patient received both treatments to assess better sensitivity. The Visual Analogue Scales (VAS) have greater metrical properties than discrete scales. Therefore, in this study a VAS was used as a more efficient substitute [39].

There was no significant difference between CULT and FMBT with regard to the evaluation of secondary caries, because no caries were observed for both study groups. The incidence of secondary caries naturally depends on the caries risk of each patient [40]. In this study, patients with high caries risk were excluded; therefore, this factor did not affect the results. Moreover, the presence of secondary caries can also be caused by the problem of marginal integrity [24]. In controversy, another study showed that the development of secondary caries is more related to the oral environment and especially saliva contamination [41]. This was not the case in this study due to rubber dam application. This can be explained by the fact that direct restorations performed with rubber dam isolation resulted in a lower failure rate than restorations performed with cotton rolls and saliva ejectors only [42]. An intriguing finding of this study is that secondary caries was absent in both techniques, it might be due to the short period of follow-up. Consequently, more attention should be given to the restoration techniques in order to avoid the formation of voids that leads to secondary caries [43].

Regarding the material fracture, excessive occlusal forces at the tooth margins can originate stress in a weak region, producing cracks and leading to small fractures [41]. However, after one year of clinical function, no significant effect of the restorative technique was observed on the fracture of the restoration.

On the other hand, the hardness of composite material plays an important role in fracture formation. Dijken et al. reported that bulk-fill composite restorations cause a continuous decrease in hardness along with an increase in depth [14]. However, according to Bucuta et al., bulk-fill composites showed hardness values similar to nanohybrid composites. All the materials reached a hardness greater than 80% after light-curing according to the manufacturer’s instructions [11]. An increase in the elastic modulus of the material reduces the effects of the internal stress caused by the polymerization shrinkage; therefore, reducing the fracture occurrence [44]. This is the case of conventional and bulk-fill composite used in this study which is characterized by a high elastic modulus. Therefore, clinicians should choose bulk-fill composites in order to reduce the undesired effects of polymerization stress while simplifying the restoration technique [18]. Additionally, this material presents less cuspal deflection when compared to the incrementally applied conventional composite [22]. In this study, a total absence of fracture was observed in both systems. This was confirmed by previous studies where the values of the fracture were similar in all the restoration groups [20,23]. These authors observed the absence of a correlation between the restoration technique and fracture resistance with the different cavity sizes. Consequently, it can be concluded that the material fracture is independent of the restoration technique.

Regarding the marginal integrity, no significant difference was observed between both techniques studied over time. Continuity between restoration and tooth structure is observed during the first year of clinical service. Several factors could affect the longevity of dental restorations, i.e., marginal integrity and leakage. If the polymerization shrinkage of the composite material exceeds the capacity of adhesive, microleakage could occur which hinders the marginal integrity [45]. While evaluating marginal integrity between the tooth and restoration, several authors showed different results depending on the application technique. Placing and light-curing a composite with 2 mm layers thickness cause the decrease in the total volumetric shrinkage of the material and the resulting stress [46]. Abbas et al. evaluated in their study a dye penetration at the margin tooth restoration, and they obtained less penetration with the incremental layering technique than with the bulk-fill restoration technique; therefore, the former gave a better marginal adaptation [47]. This finding did not match with the results of this study because the perfect marginal integrity was detected in both techniques. However, the restorations tested with bulk-fill technique did not show any microleakage; therefore, this technique seems to meet the requirements of marginal adaptation [44]. The same finding was obtained in in vitro study, in which marginal integrity of composite restorations placed with the bulk-fill technique was higher than those placed with the incremental layering technique [48].

According to the manufacturer’s instructions, a single-layer restoration technique using bulk-fill composite does not compromise the marginal adaptation of the restorations [44]. The reason for a good marginal adaptation observed with bulk-fill composite may be linked to its low polymerization stress [49]. All these results lead us to focus on bulk-fill material with a simplified modelling technique while restoring the cavity with highly configuration factor (C-factor). In high C-factor cavities, the type of composite is important for material adaptation [50]. The data of the present study confirmed that C-factor does not affect the marginal integrity. This can be explained by the fact that, in CUT, the composite is placed each time on a different wall, reducing the contact surface with the opposite walls; thus, the retraction is decreased. A low-stress composite [51,52] is applied with FMT by which the composite layer is cut into several cusps according to the tooth anatomy, decreasing the shrinkage stress and obtaining a better marginal adaptation

Concerning radiological criteria, the evaluation shows a good marginal adaptation over time for FMBT. While a gap appeared in the incremental layering restoration using the CUT in 96.7% from the first day; however, the difference was not significant between both evaluated techniques. The distinctive character of radiography in detecting defects and secondary caries can be influenced by the radiopacity of restorative material [53]. A material with adequate radiopacity may be helpful in secondary caries detection, because it would be easy to distinguish decayed tooth tissue from the restorative material. Moreover, it might facilitate the identification of defective proximal contours, voids, and inadequate marginal adaptation [54]. The radiopacity of most contemporary composites like the nanohybrid one used in this study is slightly higher than that of enamel, allowing a more precise detection of defects alongside composites [53]. The different compositions of resin monomers and the fillers characteristics (type, volume, density, and particle size) of the bulk-fill composites can affect the radiological characteristics [55]. Normally, an adequate depth of cure of bulk-fill composite is achieved by the reduction in filler content. Anyhow, the bulk-fill composite tested in this study meets the ISO requirements for radio-opacity [56]. Therefore, the clinician should choose the material with enough radio-opacity, while restoring a cavity either with CUT or FMT.

On the other hand, it was assumed that the marginal discoloration was statistically different between both techniques (*p* = 0.0244 for FMBT and 0.039 for CULT). However, at baseline and 3 months, the same result was obtained: no marginal discoloration was observed for both techniques. Over time, FMBT maintained certain stability until 12 months where a slight marginal staining between restoration and the tooth appeared. For CULT, slight marginal staining appeared after 6 months. According to the results of this study, CULT leads to more marginal discoloration than FMBT with a *p* < 0.05.

Marginal discoloration is still a problem despite the advances in monomer and the filler particle technology [57]. Due to several factors, it can be observed that surface irregularities can lead to stains and plaque retention. Physicochemical changes in the resin structure, conversion rate, and water absorption may also facilitate staining susceptibility [51]. Yazici et al. also suggested that marginal discoloration may be affected by the type of adhesive used [49]. However, this is not the only factor responsible for the discoloration because in this study the same adhesive is used in both types of restoration. The color stability of resin composites can be related to the material properties such as: resin matrix, filler technology, interface matrix-filler, degree of polymerization, and the application technique [58]. The resistance of the material to discoloration depends on the monomer content and surface roughness more than on the size of the filler particles [59].

It was claimed that the hybrid resin composites are difficult to polish and maintain. The clinical reality is that it is possible to obtain ideal surfaces with such materials, and due to their composition, they provide optimal stability over time [60,61]. This could not be in agreement with this study because, after 6 months, the nanohybrid composite used in CULT showed a slight coloration despite the best polish and surface smoothness obtained at early stages. Thus, one should bear in mind that a slight surface discoloration does not require additional treatment and can be disregarded, which is the case in this study.

However, the case of more significant discoloration involves the penetration of debris containing cariogenic bacteria [41]. Consequently, this generates plaque accumulation, secondary caries, abrasiveness, restoration marginal discoloration, and wear kinetics distribution. Cavities restored with a single layer using bulk-fill composite resulted in significantly better clinical results than conventional composites in terms of marginal discoloration [49]. This supported the findings of this study as bulk-fill posterior composite is promising.

The conventional incremental layering technique is highly desired to reduce problems generated by the polymerization stress [2,12]. Consequently, the posterior teeth restoration technique must guarantee a correct adaptation of resin composite, in particular at the marginal limits of the cavity, correct reconstruction of the tooth anatomy, a fracture and wear resistance, a reduction in shrinkage stress, and thus longevity of the restoration. These factors meet the requirement of the newly introduced FMBT as simple, reproducible, and less technique sensitive method. This technique does not require attention to details during the placement of each layer in extended or deep cavities, carrying an implicit risk of incorporating impurities or air bubbles between layers. All of this decreases the treatment time, mainly when compared to CULT. Furthermore, it has been noticed that after 3 years of clinical evaluation, the bulk-fill composite showed an excellent behavior when compared to its use in an incremental layering technique, mainly when associated to etch-and-rinse adhesives [62]. This study exhibited that in high C-factor cavity, with an etch-and-rinse adhesive, it is possible to successfully work with a bulk-fill material using the FMT. Moreover, decreased shrinkage stress of bulk-fill composites was observed when compared to hybrid composites. However, in a 3 year clinical study, a bulk-fill restoration technique showed similar results to the incremental layering technique. These results seem promising, but further research should validate a long-term result [63].

The limitations of this study should be taken into consideration. First of all, studies of one or two year follow-up might provide useful information about the clinical performance of the newly introduced modelling technique: FMBT, but an accurate evaluation of its longevity still requires long-term follow-up time (5 years to over 10 years). Furthermore, a comparison between FMT and CUT both with nanohybrid composite should be researched in future clinical studies. Next, further studies are needed testing more dental adhesives and resin composites to demonstrate broad comparison between different materials. In this study, the restoration materials were combined with the recommended adhesive of the same manufacturer [64]. In addition, high-risk patients were excluded. However, these challenges are encountered by dentists in daily practice and require a scientific background to guide them in decision making on the adoption or rejection of new materials and techniques [65]. Moreover, caution should be considered for material-, patient-, and dentist-related factors since the longevity of restorations could be affected by these variables [66]. Lastly, few participants were involved in this study; therefore, collecting information from numerous patients should be researched clinically in future to confirm the current preliminary results.

## 5. Conclusions

Within the limitations of this study, clinical parameters of restorations placed using CULT and FMBT were comparable. However, it should be noted that FMBT presented a significantly lower marginal discoloration in comparison to CULT. Consequently, the null hypothesis tested was partially accepted.

Moreover, clinicians could find the procedure of FMBT easy and feasible. Additionally, fewer layers of bulk-fill composite might be needed as compared to the traditional CULT in the posterior teeth.

## Figures and Tables

**Table 1:** Manufacturer and composition of the materials used for performing the restorations.

Material	Type	Manufacturer	Composition
Filtek Z250XT	Nanohybrid composite	3M ESPE, St. Paul, MN, USA	Bis-GMA; UDMA; Bis-EMA; PEGDMA and TEGDMA. Surface-modified zirconia/silica with a median particle size of approximately 3 μm or less. Nonagglomerated/nonaggregated 20 nm surface-modified silica particles. The filler loading is 82% by weight (68% by volume)
Filtek bulk-fill Posterior Restorative	Bulk-fill composite	3M ESPE, St. Paul, MN, USA	AUDMA; UDMA; and 1, 12-dodecane-DMA 20 nm silica filler, a non-agglomerated nonaggregated 4–11 nm zirconia filler, an aggregated zirconia/silica cluster filler (comprised of 20 nm silica and 4–11 nm zirconia particles), and a ytterbium trifluoride filler consisting of agglomerate 100 nm particles. Inorganic filler loading is about 76.5% by weight (58.4% by volume)
Scotchbond™ Universal Etchant	Low-viscosity etching gel	3M ESPE, St. Paul, MN, USA	34% H_3_PO_4_, water, synthetic amorphous silica, polyethylene glycol, aluminum oxide
Adper™ Single Bond	Two-step etch-and-rinse adhesive	3M ESPE, St. Paul, MN, USA	Bis-GMA; HEMA, dimethacrylates, polyalkenes acid copolymer, initiators, water, and ethanol

PEGDMA: Polyethylene glycol dimethacrylate; TEGDMA: triethylene glycol dimethacrylate; Bis-EMA: ethoxylatedbisphenol A dimethacrylate; UDMA: urethane dimethacrylate; BisGMA: bisphenol A-glycidyl methacrylate; and HEMA: 2-hydroxyethyl methacrylate.

**Table 2:** Federation Dentaire Internationale (FDI) criteria used to assess the biological, functional, and aesthetic properties of restorations.

Outcome Variables and Methods for the Evaluation of Restorations		Clinically Excellent	Clinically Good	Clinically Sufficient	Clinically Unsatisfactory	Clinically Poor
Biological Properties	Sensitivity	Absence of pain	Low pain	Moderate pain	Intense pain	Extreme pain
	Secondary caries	No secondary caries	Small and localized. Demineralization area	Larger areas of demineralization. Only preventive measures necessary	Caries with cavitation and suspected undermining caries. Localized and accessible can be repaired	Deep secondary caries or exposed dentine that is not accessible for repair of restoration
Functional Properties	Fracture	Restoration in place, no fracture	Small hairline crack	Two or more or larger hairline cracks and/or chipping (not affecting the marginal integrity or approximal contact)	Material chip fractures which damage marginal quality and/or approximal contacts	(Partial or complete) loss of the restoration or multiple fractures
	Marginal integrity	Continuity between restoration/tooth	Marginal gap (<150 μm), white lines. Small marginal fracture removable by polishing	Gap < 250 μm not removable. Several small marginal fractures	Gap > 250 μm or dentine/base exposed. Severe marginal fractures	Restoration (complete or partial) is loose but in situ. Generalized major gaps or irregularities
	Radiographic examination	No pathology, harmonious transition between restoration and tooth	Acceptable material excess present. Positive/negative step present at margin < 150 μm	Marginal gap < 250 μm. Negative steps visible < 250 μm. No adverse effects noticed. Poor radiopacity of filling material	Marginal gap > 250 μm. Material excess accessible but not removable. Negative steps > 250 μm and reparable	Secondary caries, large gaps, large overhangs. Apical pathology. Fracture/loss of restoration or tooth
Aesthetic Property	Marginal discoloration	No marginal staining	Minor staining, easily removable	Moderate staining between restoration and tooth	Surface staining recognizable from speaking distance. Or severe localized marginal staining not removable by polishing	Significant (detectable) marginal staining between restoration and tooth

**Table 3:** Assessment of sensitivity for restored teeth with FMBT and CULT over time (*p* > 0.05).

Sensitivity		Absence of Pain	Low Pain	Moderate Pain	Intense Pain	Extreme Pain
FMBT	Baseline	30 (100.0%)	0 (0.0%)	0 (0.0%)	0 (0.0%)	0 (0.0%)
	3 months	29 (96.7%)	1 (3.3%)	0 (0.0%)	0 (0.0%)	0 (0.0%)
	6 months	28 (93.3%)	2 (6.7%)	0 (0.0%)	0 (0.0%)	0 (0.0%)
	12 months	28 (93.3%)	2 (6.7%)	0 (0.0%)	0 (0.0%)	0 (0.0%)
CULT	Baseline	30 (100.0%)	0 (0.0%)	0 (0.0%)	0 (0.0%)	0 (0.0%)
	3 months	28 (93.3%)	2 (6.7%)	0 (0.0%)	0 (0.0%)	0 (0.0%)
	6 months	26 (86.7%)	2 (6.7%)	2 (6.7%)	0 (0.0%)	0 (0.0%)
	12 months	26 (86.7%)	2 (6.7%)	2 (6.7%)	0 (0.0%)	0 (0.0%)

**Table 4:** Assessment of secondary caries for teeth restored with FMBT and CULT over time (*p* > 0.05).

Secondary Caries		No Secondary Caries	Small and Localized. Demineralization Area	Larger Areas of Demineralization. Only Preventive Measures Necessary	Caries with Cavitation and Suspected Undermining Caries. Localized and Accessible Can Be Repaired	Deep Secondary Caries or Exposed Dentine that Is Not Accessible for Repair of Restoration
FMBT	Baseline	30 (100.0%)	0 (0.0%)	0 (0.0%)	0 (0.0%)	0 (0.0%)
	3 months	30 (100.0%)	0 (0.0%)	0 (0.0%)	0 (0.0%)	0 (0.0%)
	6 months	30 (100.0%)	0 (0.0%)	0 (0.0%)	0 (0.0%)	0 (0.0%)
	12 months	30 (100.0%)	0 (0.0%)	0 (0.0%)	0 (0.0%)	0 (0.0%)
CULT	Baseline	30 (100.0%)	0 (0.0%)	0 (0.0%)	0 (0.0%)	0 (0.0%)
	3 months	30 (100.0%)	0 (0.0%)	0 (0.0%)	0 (0.0%)	0 (0.0%)
	6 months	30 (100.0%)	0 (0.0%)	0 (0.0%)	0 (0.0%)	0 (0.0%)
	12 months	30 (100.0%)	0 (0.0%)	0 (0.0%)	0 (0.0%)	0 (0.0%)

**Table 5:** Fracture of restored teeth with FMBT and CULT over time (*p* > 0.05).

Fracture		Restoration in Place, No Fracture	Small Hairline Crack	Two or More or Larger Hairline Cracks and/or Chipping (Not Affecting the Marginal Integrity or Approximal Contact)	Material Chip Fractures Which Damage Marginal Quality and/or Approximal Contacts	(Partial or Complete) Loss of the Restoration or Multiple Fractures
FMBT	Baseline	30 (100.0%)	0 (0.0%)	0 (0.0%)	0 (0.0%)	0 (0.0%)
	3 months	30 (100.0%)	0 (0.0%)	0 (0.0%)	0 (0.0%)	0 (0.0%)
	6 months	30 (100.0%)	0 (0.0%)	0 (0.0%)	0 (0.0%)	0 (0.0%)
	12 months	30 (100.0%)	0 (0.0%)	0 (0.0%)	0 (0.0%)	0 (0.0%)
CULT	Baseline	30 (100.0%)	0 (0.0%)	0 (0.0%)	0 (0.0%)	0 (0.0%)
	3 months	30 (100.0%)	0 (0.0%)	0 (0.0%)	0 (0.0%)	0 (0.0%)
	6 months	30 (100.0%)	0 (0.0%)	0 (0.0%)	0 (0.0%)	0 (0.0%)
	12 months	30 (100.0%)	0 (0.0%)	0 (0.0%)	0 (0.0%)	0 (0.0%)

**Table 6:** Assessment of marginal integrity of restored teeth with FMBT and CULT over time (*p* > 0.05).

Marginal Integrity		Continuity between Restoration/Tooth	Marginal Gap (<150 μm), White Lines. Small Marginal Fracture Removable by Polishing	Gap < 250 μm Not Removable. Several Small Marginal Fractures	Gap > 250 μm or Dentine/Base Exposed. Severe Marginal Fractures	Restoration (Complete or Partial) Is Loose but in Situ. Generalized Major Gaps or Irregularities
FMBT	Baseline	30 (100.0%)	0 (0.0%)	0 (0.0%)	0 (0.0%)	0 (0.0%)
	3 months	30 (100.0%)	0 (0.0%)	0 (0.0%)	0 (0.0%)	0 (0.0%)
	6 months	30 (100.0%)	0 (0.0%)	0 (0.0%)	0 (0.0%)	0 (0.0%)
	12 months	30 (100.0%)	0 (0.0%)	0 (0.0%)	0 (0.0%)	0 (0.0%)
CULT	Baseline	30 (100.0%)	0 (0.0%)	0 (0.0%)	0 (0.0%)	0 (0.0%)
	3 months	30 (100.0%)	0 (0.0%)	0 (0.0%)	0 (0.0%)	0 (0.0%)
	6 months	30 (100.0%)	0 (0.0%)	0 (0.0%)	0 (0.0%)	0 (0.0%)
	12 months	30 (100.0%)	0 (0.0%)	0 (0.0%)	0 (0.0%)	0 (0.0%)

**Table 7:** Marginal adaptation of teeth restored with FMBT and CULT over time (*p* > 0.05).

Marginal Adaptation		No Pathology, Harmonious Transition between Restoration and Tooth	Acceptable Material Excess Present. Positive/Negative Step Present at Margin < 150 μm	Marginal Gap < 250 μm. Negative Steps Visible < 250 μm. No Adverse Effects Noticed. Poor Radiopacity of Filling Material	Marginal Gap > 250 μm. Material Excess Accessible but Not Removable. Negative Steps > 250 μm and Reparable	Secondary Caries, Large Gaps, Large Overhangs. Apical Pathology. Fracture/Loss of Restoration or Tooth
FMBT	Baseline	30 (100.0%)	0 (0.0%)	0 (0.0%)	0 (0.0%)	0 (0.0%)
	3 months	30 (100.0%)	0 (0.0%)	0 (0.0%)	0 (0.0%)	0 (0.0%)
	6 months	30 (100.0%)	0 (0.0%)	0 (0.0%)	0 (0.0%)	0 (0.0%)
	12 months	30 (100.0%)	0 (0.0%)	0 (0.0%)	0 (0.0%)	0 (0.0%)
CULT	Baseline	29 (96.7%)	0 (0.0%)	1 (3.3%)	0 (0.0%)	0 (0.0%)
	3 months	29 (96.7%)	0 (0.0%)	1 (3.3%)	0 (0.0%)	0 (0.0%)
	6 months	29 (96.7%)	0 (0.0%)	1 (3.3%)	0 (0.0%)	0 (0.0%)
	12 months	29 (96.7%)	0 (0.0%)	1 (3.3%)	0 (0.0%)	0 (0.0%)

**Table 8:** Marginal staining of teeth restored with FMBT and CULT over time (*p* < 0.05).

Marginal Discoloration No Marginal Staining			Minor Staining, Easily Removable	Moderate Staining between Restoration and Tooth	Surface Staining Recognizable from Speaking Distance. or Severe Localized Marginal Staining Not Removable by Polishing	Significant (Detectable) Marginal Staining between Restoration and Tooth
FMBT	Baseline	30 (100.0%)	0 (0.0%)	0 (0.0%)	0 (0.0%)	0 (0.0%)
	3 months	30 (100.0%)	0 (0.0%)	0 (0.0%)	0 (0.0%)	0 (0.0%)
	6 months	30 (100.0%)	0 (0.0%)	0 (0.0%)	0 (0.0%)	0 (0.0%)
	12 months	28 (93.3%)	2 (6.7%)	0 (0.0%)	0 (0.0%)	0 (0.0%)
CULT	Baseline	30 (100.0%)	0 (0.0%)	0 (0.0%)	0 (0.0%)	0 (0.0%)
	3 months	30 (100.0%)	0 (0.0%)	0 (0.0%)	0 (0.0%)	0 (0.0%)
	6 months	27 (90.0%)	3 (10.0%)	0 (0.0%)	0 (0.0%)	0 (0.0%)
	12 months	26 (86.7%)	4 (13.3%)	0 (0.0%)	0 (0.0%)	0 (0.0%)

## Data Availability

The data presented in this study are available on request from the corresponding author.
